# Drought-induced susceptibility for *Cenangium ferruginosum* leads to progression of *Cenangium*-dieback disease in *Pinus koraiensis*

**DOI:** 10.1038/s41598-018-34318-6

**Published:** 2018-11-06

**Authors:** Minji Ryu, Ratnesh Chandra Mishra, Junhyun Jeon, Sun Keun Lee, Hanhong Bae

**Affiliations:** 1 Department of Biotechnology, Yeungnam University, Gyeongsan, Gyeongbuk 38541 Republic of Korea; 2 Division of Forest Insect Pests and Diseases, National Institute of Forest Science, Seoul, 02455 Republic of Korea

## Abstract

Recently, the occurrence of “*Cenangium*-dieback” has been frequent and devastating. *Cenangium*-dieback is caused by an endophytic fungus *Cenangium ferruginosum* in stressed pine trees. Progression of the disease in terms of molecular interaction between host and pathogen is not well studied and there is a need to develop preventive strategies. Thus, we simulated disease conditions and studied the associated transcriptomics, metabolomics, and hormonal changes. *Pinus koraiensis* seedlings inoculated with *C*. *ferruginosum* were analyzed both under drought and well-watered conditions. Transcriptomic analysis suggested decreased expression of defense-related genes in *C*. *ferruginosum*-infected seedlings experiencing water-deficit. Further, metabolomic analysis indicated a decrease in the key antimicrobial terpenoids, flavonoids, and phenolic acids. Hormonal analysis revealed a drought-induced accumulation of abscisic acid and a corresponding decline in the defense-associated jasmonic acid levels. Pathogen-associated changes were also studied by treating *C*. *ferruginosum* with metabolic extracts from pine seedlings (with and without drought) and polyethylene glycol to simulate the effects of direct drought. From RNA sequencing and metabolomic analysis it was determined that drought did not directly induce pathogenicity of *C*. *ferruginosum*. Collectively, we propose that drought weakens pine immunity, which facilitates increased *C*. *ferruginosum* growth and results in conversion of the endophyte into the phytopathogen causing dieback.

## Introduction

Pine species are distributed in most regions of the northern hemisphere. Many species have been introduced to temperate and subtropical regions of the southern hemisphere as well. Pines have high economical value and are grown as timber. As pines are evergreen, they are also commonly cultivated as ornamental plants in parks and gardens. There are a number of pine diseases that affect the growth and survival of different pine species. One of the deadliest diseases is pine-dieback, which is caused by a number of fungal pathogens, e.g., *Cenangium ferruginosum*, *Mycosphaerella pini*, *Gremmeniella abietina*, and *Shaeropsis sapinea*, among others^1^. Recently, the outbreak of *Cenangium*-dieback (caused by *C*. *ferruginosum*) has been frequent and devastating^1,2^. *C*. *ferruginosum* is reportedly an endophytic fungus that lives as a biotroph in healthy pine trees and is considered a primary decomposer of weakened and dying trees^2^. However, under stressful conditions such as drought, extreme cold, unusual warm winters, and wounding, the endophytic fungus turns pathogenic, leading to detrimental consequences in pine trees^3^. The disease is first marked by the appearance of needle necrosis. Subsequently, it spreads causing damage to twigs, branches, and eventually the whole tree (hence the name “dieback”)^3^. Currently, *Cenangium*-dieback cases are not frequently reported worldwide; however, a number of pine species were seriously affected by this disease in different regions of Korea in the spring of 2007 and 2009^3^. *C*. *ferruginosum* infects and damages different pine species in Korea, namely *Pinus densiflora*, *P*. *thunbergii*, and *P*. *koraiensis*^2^, but strong pathogenicity of the fungus was observed on *P*. *koraiensis*. Importantly, a recent outbreak of *Cenangium*-dieback was reported in early spring 2012 from several regions of Slovakia^1^, suggesting a wide geographical spread in the occurrence of the disease, which is alarming.

The defense system in pine species is well equipped with various molecular arsenals to combat pathogen attack. Most of them are considered to be mediated by phytohormone-dependent pathways. Necrotrophic fungi have been reported to largely activate the jasmonic acid (JA)-dependent defenses in plants^4^. Once activated, these pathways lead to the production of pathogenesis-related (PR) proteins and induction of flavonoid biosynthetic pathways, thereby inducing the synthesis of secondary metabolites that act as antimicrobial agents in plant-microbe interactions^5^. Needles of pine trees are well known to have antimicrobial effects by virtue of different terpenoids, flavonoids, and phenolic acids that kill microbial cells. The major pine defenses against fungi are regulated by specialized chemicals namely terpenoids of oleoresin and phenolics^6,7^. The role of terpenoids in tree-fungus interactions and associated biochemical pathways are characterized in conifer species^8–10^. Diverse and variable terpenoids of conifers exist as monoterpenes, sesquiterpenes, and diterpenes, including the diterpene resin acids. Oleoresins, composed mostly of monoterpenes and diterpene resin acids, provide both physical as well as chemical defenses against microbes^11^. In general, pines produce and store large amount of terpenoids. Over 40,000 different terpenoids have been characterized so far in plants^12^. Among them, several compounds are produced by primary metabolism, including some phytohormones, such as abscisic acid (ABA). However, the majority of them are secondary metabolites, which are known to have defensive roles and are species specific^12^. Members of the *Pinus* genus are known to accumulate high levels of terpenoids, especially essential oils and resins, as defense compounds^6,8,13^. Terpenoids quantities and composition in the oleoresin and volatile emissions can vary dramatically according to the plant defensiveness, environmental conditions, and fungal attacks^8,14^. Besides terpenoids, the other important groups of bioactive compounds are flavonoids and phenolic acids. Flavonoids show diverse structure and a broad range of biological activities, with free aglycones and glycoside forms^15^. Phenolic acids, phenylpropanoids with an attached three carbon side chain and aromatic ring, exist in nature and have free and bound forms as esters and glycosides. The flavonoids and phenolic acids have protective roles against both microbial attack and environmental stresses^15,16^.

Defense mechanisms of pines against *C*. *ferruginosum* are considered to be complex and differ from those that are effective against other endophytic biotrophs. Studies addressing the conversion mechanism of an endophyte into a phytopathogen are largely lacking and thus the scientific knowledge in this area is still in its infancy. In the wake of the severity and continuing trend of the occurrence of *Cenangium*-dieback, which has been frequent and covered a large geographical area, the development of preventive strategies is urgently required. To do so, however, it is important to first understand the molecular interaction between the host and the pathogen to shed light on the mechanism of disease progression. Necessitated by this, we studied the progression of *Cenangium*-dieback at the molecular level from the perspective of both the host and the pathogen. Because *C*. *ferruginosum* exhibits high pathogenicity in *P*. *koraiensis*, making it an important case to study, we chose this pine species as a model in our work. In our study, we investigated the pathogenicity of *C*. *ferruginosum* in *P*. *koraiensis* with and without drought stress treatment to elucidate the mechanisms of dieback progression. The interaction between the *P*. *koraiensis* and *C*. *ferruginosum* was studied at the transcriptome level, using an RNA sequencing (RNA-seq) approach. Additionally, the alteration in compounds (terpenoids, flavonoids, and phenolics) related to immunity in *P*. *koraiensis* induced by *C*. *ferruginosum* and subsequent drought was also examined. The effect of drought on the pathogenicity of the fungus was also taken into consideration. Our results suggested that drought weakens *P*. *koraiensis* immunity against *C*. *ferruginosum*, by virtue of reduced expression of the pine’s defense genes and secondary metabolites. This facilitates increased fungal growth leading to the development of *Cenangium*-dieback disease. We believe that our study is a major advance in this field, which will accelerate/facilitate further studies. This will eventually lead to the design of fruitful combative strategies.

## Results

### *Cenangium*-dieback outbreak in *P. koraiensis*

**Figure 1:**
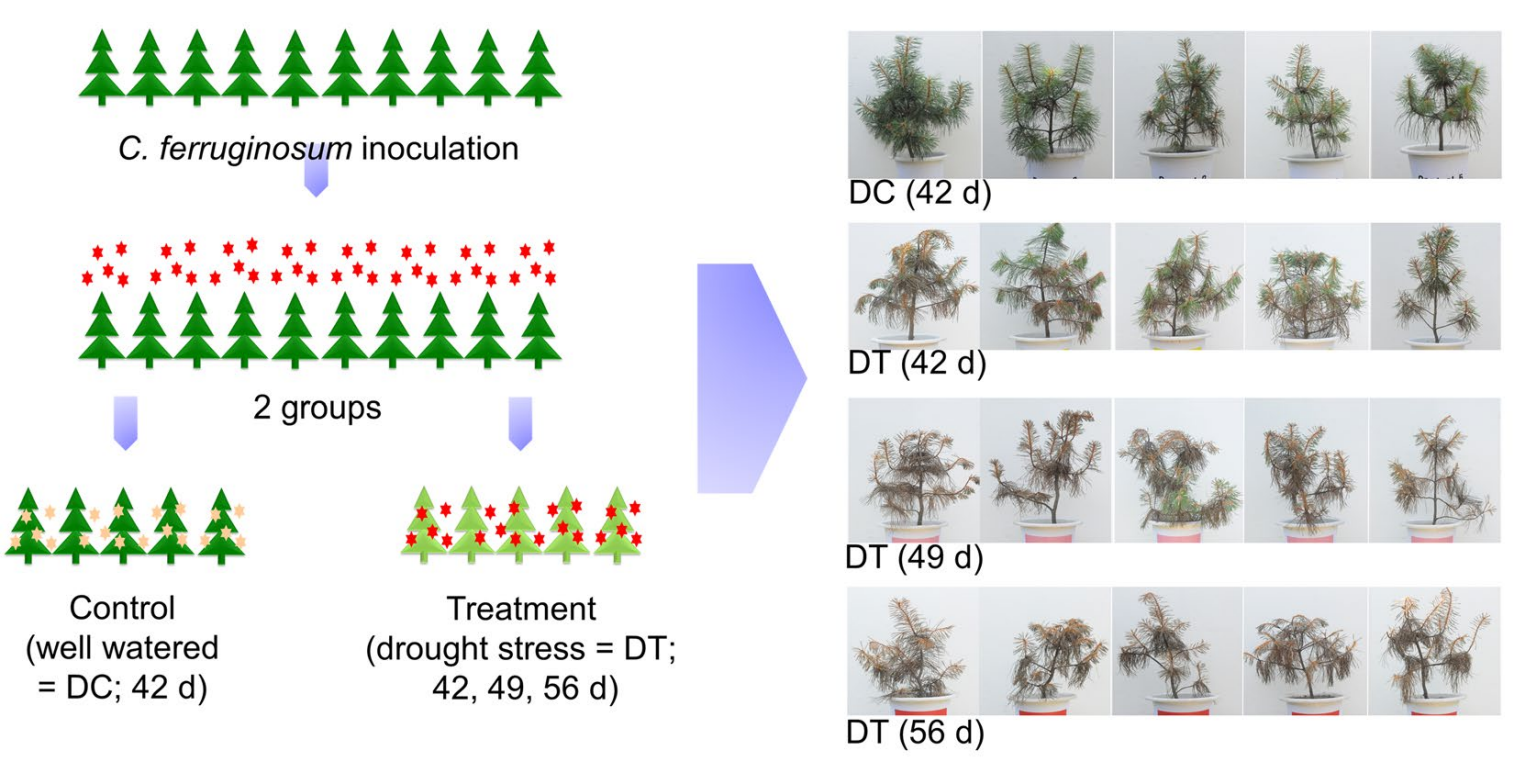
Experimental scheme and appearance of *Cenangium*-dieback in *Pinus koraiensis* after drought stress. Pine seedlings (5-year old) were infected with *Cenangium ferruginosum* and divided into two groups. Pine seedlings were grown under well-watered (DC) and drought stress (DT) conditions for 42, 49, and 56 d post infection. Development of *Cenangium*-dieback occurred in pine seedlings by progressing drought stress.

The pine seedlings infected by *C*. *ferruginosum* and grown under normal water supply for 42 d visibly appeared to be healthy (Fig. 1), whereas infected seedlings that experienced drought for 42, 49, and 56 d showed overall necrotic symptoms (Fig. 1). In case of non-infected seedlings, the needles showed slight wilting without necrotic symptoms after 42 d of drought as compared to the control (data not shown).

### Generation of RNA-sequencing *de novo* assembly of *C*. *ferruginosum*

**Figure 2:**
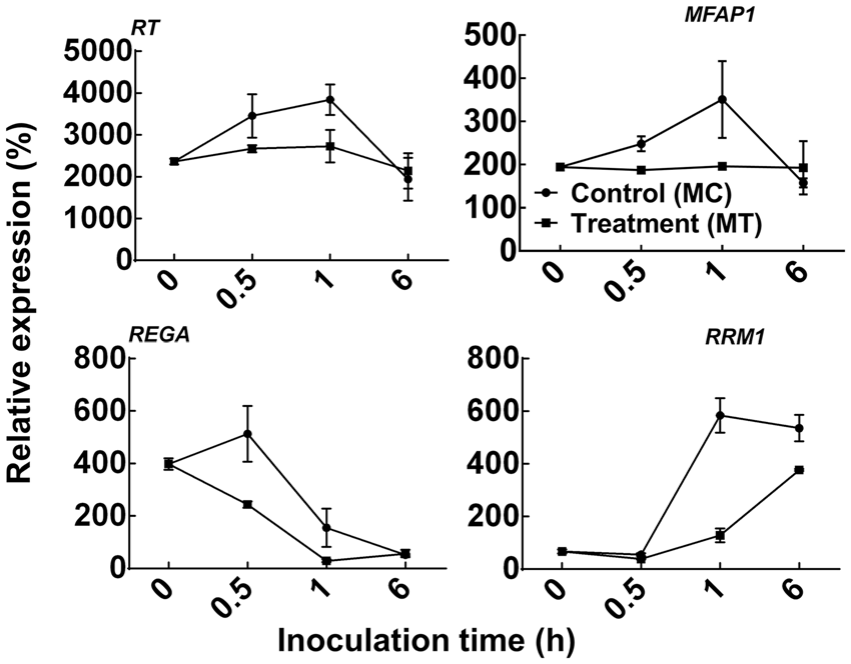
Expression analysis of four *Cenangium ferruginosum* transcripts after treatment with metabolic extracts of *Pinus koraiensis*. Transcript level was quantified for four *C*. *ferruginosum* transcripts encoding reverse transcriptase (*RT*), microfibrillar associated protein 1 (*MFAP1*), DNA binding response regulator PrrA (*REGA*), and ribonucleotide reductase catalytic subunit M1 (*RRM*1) after treatment with metabolic extracts of pine seedlings in MSB liquid culture. The relative expression values were obtained by qRT-PCR normalized against the *C*. *ferruginosum ACTIN g*ene and are shown as the averages ± standard error obtained from three biological replications. In the case of the control (MC), metabolic extracts of pine seedlings grown under optimal water supply were added, whereas in the case of the treatment (MT), metabolic extracts obtained from drought-stressed pine seedlings were added.

Sets of *C*. *ferruginosum* transcriptomes were prepared under different biological conditions, representing growth and pathogenicity *in vitro*. The fungal response to artificial drought was assessed through *in vitro* polyethylene glycol (PEG) treatment. Fungal response to treatments with metabolic extracts of healthy and drought-stressed pine seedlings was also screened. The goal was to develop a *de novo* RNA-seq assembly and reveal if drought has any effect on the growth and pathogenicity of the fungus. The growth of the fungus was unaffected after treatment with different concentrations of PEG (Supplementary Fig. S1). Also, no remarkable change in the transcriptomic profiles of the fungus treated with pine metabolites, as well as PEG, was noted. Expression patterns were validated by quantitative reverse-transcription PCR (qRT-PCR) analysis for the four growth-related fungal genes encoding reverse transcriptase (*RT*), microfibrillar associated protein 1 (*MFAP1*), DNA-binding response regulator PrrA (*REGA*), and ribonucleotide reductase catalytic subunit M1 (*RRM1*), in fungal samples treated with pine metabolites from healthily growing and drought stressed pine seedlings (Fig. 2). The expression of genes related to growth and protection against damage was induced in *C*. *ferruginosum* treated with metabolites from drought stressed pine trees as compared to that of the well-watered trees. A strong correlation between the results of qRT-PCR and RNA-seq was noted.

### Analysis of *P*. *koraiensis* transcriptomic map in response to drought stress post *C*. *ferruginosum* infection

**Figure 3:**
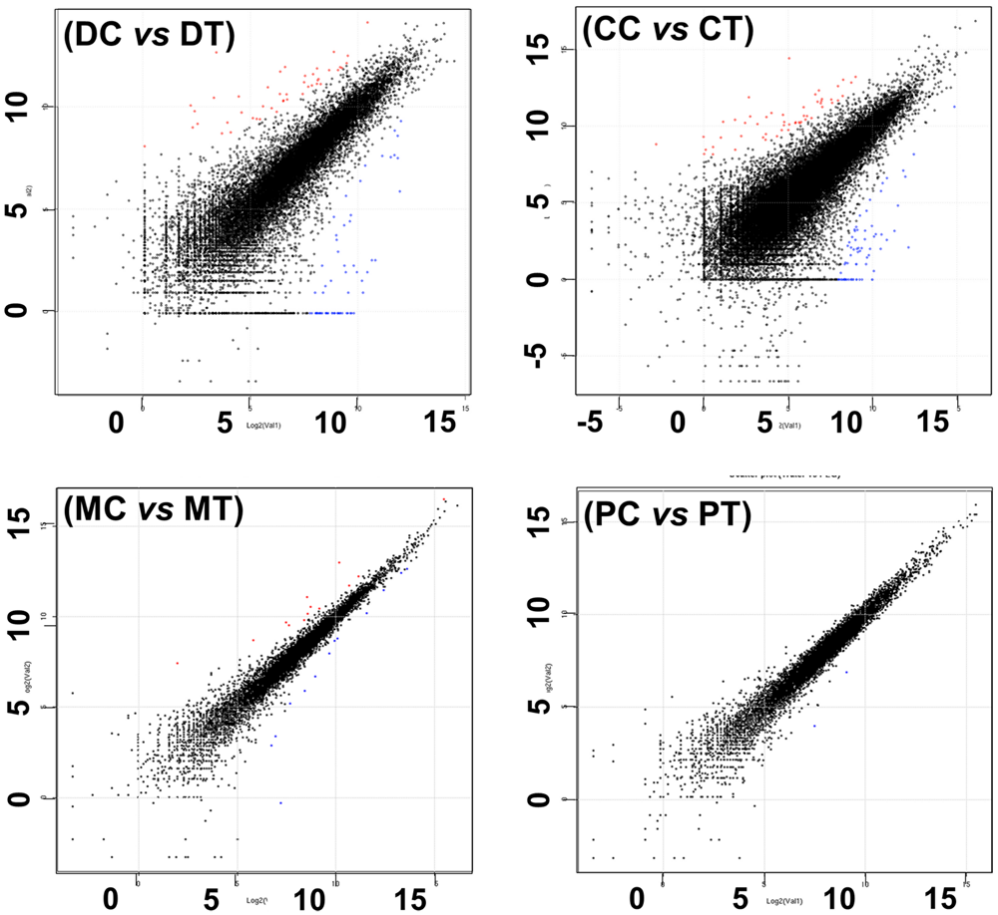
Global expression analysis of *Pinus koraiensis* and *Cenangium ferruginosum* transcripts. The Reads Per Kilobase per Million (RPKM) mapped reads values for all unigenes were plotted for the control and treatment samples. Unigene significant changes (more than 2-fold) are in red and blue for up- and down-regulation, respectively. The black dots indicate no significant difference. Scatter plots present the values of log2 for each unigene for the control (X-axis) versus treatment samples (Y-axis). Refer to Table 1 for information regarding the abbreviation used.

Two sets of RNA-seq were performed to reveal the gene expression changes in pine seedlings: two representative pine seedlings under well-watered and drought conditions for 42 d post *C*. *ferruginosum* infection. From analysis using pine reference, a total of 3,482,841,200 paired reads were generated (Table 1). Samples from the pine seedlings growing with and without water for 42 d were represented by 1,528,354,456 (93.8%) and 1,738,816,576 (93.8%) clean paired reads, respectively. For both RNA-seq libraries, 86,125,157 base pairs (bps) were mapped with genome references available for other pine species and 91,125 unigenes were identified. A remarkable difference in the expression of transcripts in seedlings experiencing drought post infection and the respective control was noted from the scatter plot (Fig. 3). The thick line in the scatter plot indicates a significant difference between the treatment and control, whereas the thin line indicates the lack of a significant difference between the treatment and control. A strong correlation (r > 0.846) between the genes expressed in infected seedlings treated with drought stress and watered optimally (control) was found.

**Table 1:** Sequencing metrics of the 8 RNA-seq libraries.

Name	Source	Treatment	Total paired reads	Clean paired reads	Mapping base pairs	No. of unigene	AU^i^
DC^a^	Pine tree	*Cenangium* No drought	1,628,657,000	1,528,354,456 (93.8%)	86,125,157	91,125	945
DT^b^		*Cenangium* Drought	1,854,184,200	1,738,816,576 (93.8%)			
CC^c^	*Cenangium* in pine tree	*Cenangium* No drought	2,363,872,000	2,233,447,377 (94.5%)	99,482,697	111,849	889
CT^d^		*Cenangium* Drought	2,604,394,000	2,451,341,847 (94.1%)			
MC^e^	*Cenangium*	Pine metabolite without drought	1,410,540,400	1,330,739,479 (94.3%)	43,557,605	26,603	1,637
MT^f^		Pine metabolite with drought	1,395,221,600	1,308,616,813 (93.8%)			
PC^g^	*Cenangium*	Water	1,372,241,200	1,289,821,645 (94.0%)			
PT^h^		PEG	1,103,343,800	1,049,283,477 (95.1%)			

^a^DC indicates pine tree data in *P*. *koraiensis* grown under well-watered condition for 42 d post *C*. *ferruginosum* inoculation.

^b^DT indicates pine tree data in *P*. *koraiensis* exposed to drought for 42 d post *C*. *ferruginosum* inoculation.

^c^CC indicates *C*. *ferruginosum* data in *P*. *koraiensis* grown under well-watered condition for 42 d post *C*. *ferruginosum* inoculation.

^d^CT indicates *C*. *ferruginosum* data in *P*. *koraiensis* exposed to drought for 42 d post *C*. *ferruginosum* inoculation.

^e^MC indicates *C*. *ferruginosum* data in *C*. *ferruginosum* treated with metabolic extract of healthily growing *P*. *koraiensis* for 1 h.

^f^MT indicates *C*. *ferruginosum* data in *C*. *ferruginosum* treated with metabolic extract of drought-stressed *P*. *koraiensis* for 1 h.

^g^PC indicates *C*. *ferruginosum* data in *C*. *ferruginosum* treated with distilled water for 1 h.

^h^PT indicates *C*. *ferruginosum* data in *C*. *ferruginosum* treated with 5% PEG 6000 for 1 h.

^i^AU indicates average length of unigene (bp).

To decipher *C*. *ferruginosum* genes, which were differentially expressed in pine seedlings as a result of infection under drought, the above RNA-seq data sets were analyzed against *C*. *ferruginosum* reference. A total of 4,968,266,000 paired reads (Table 1) were generated from samples from the infected seedlings, and those growing with and without water for 42 d were represented by 2,233,447,377 (94.5%, CC) and 2,451,341,847 (94.1%, CT) clean paired reads, respectively. A remarkable difference in the expression of transcripts in seedlings experiencing drought post-infection and their respective control was noted from the scatter plot (Fig. 3).

**Figure 4:**
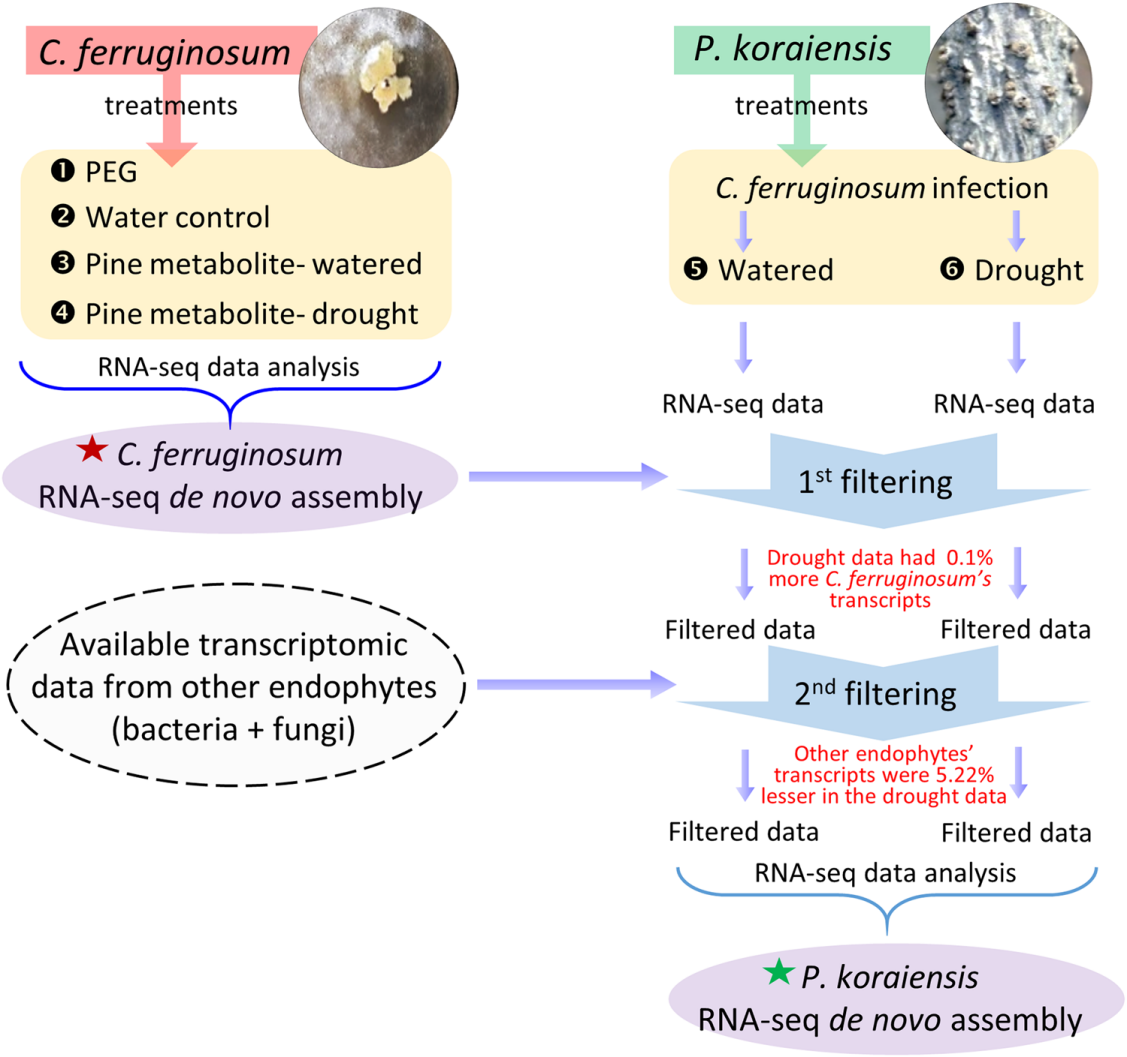
Model summarizing the RNA-seq workflow for the generation of RNA-seq *de novo* assembly of *Pinus koraiensis*. Total 6 different sets of RNA-seq data that were generated in the work are indicated by numbers (1–6). RNA-seq data from the well-watered and drought-exposed infected seedlings were obtained (5 & 6). From the obtained data, *Cenangium ferruginosum* reads were screened out by using the RNA-seq *de novo* assembly of *C*. *ferruginosum* generated separately (indicated by the red star). The filtered data then underwent another screening process to eliminate the reads from other endophytic bacteria/fungi (available on public databases). Finally, after two rounds of filtering, the RNA-seq *de novo* assembly of *P*. *koraiensis* was obtained (indicated by the green star).

Expectedly, the RNA-seq data from infected seedlings contained reads, besides those from the pine trees, including reads from *C*. *ferruginosum* and other endophytic bacteria/fungus (supposedly less compared to mountain-grown seedlings, as they were grown in pots maintained in a glasshouse) growing within the pine seedlings. To generate the RNA-seq *de novo* assembly of the *P*. *koraiensis*, the RNA-seq data underwent two filtering steps (Fig. 4). First, the *C*. *ferruginosum* reads were identified using RNA-seq *de novo* assembly of *C*. *ferruginosum* and filtered out. Thereafter, the filtered data was screened for the reads from other bacterial/fungal contaminants/endophytes. A total of 99,482,697 bps was mapped to the *C*. *ferruginosum* reference (Table 1). The filtered *de novo* assemblies of pine tree were then analyzed for transcript expression. Of the 91,125 pine tree unigenes identified above, 143 unigenes were noted to be up-regulated and 249 unigenes were down-regulated, totaling 392 differentially expressed genes (q-value < 0.05) in the drought-exposed infected pine seedlings.

### Transcripts of *C. ferruginosum* in *P. koraiensis*

As discussed above, approximately 99 million bp of the reads obtained from infected seedlings were mapped to the *C*. *ferruginosum* reference assembly (Table 1). A total of 111,849 *C*. *ferruginosum* unigenes were detected in infected seedlings growing both under optimal water and drought stress. Out of the 111,849 unigenes, 115 unigenes were up-regulated and 232 unigenes were down-regulated, totaling 347 differentially expressed genes (q-value < 0.05), in drought experiencing seedlings (Table 1).

Interestingly, there was a 0.1% increase in the reads (from 7.49% to 7.59%) that mapped to *C*. *ferruginosum* genes when control seedlings (well-watered infected seedlings) were compared with treated seedlings (infected seedlings experiencing drought) (Fig. 4). Contrastingly, however, reads from other endophytes declined from 23.95% to 18.73% (5.22% decrease) in the above-mentioned samples. For the ease of understanding the entire scheme is depicted as a flow chart in Fig. 4.

### Characterization of the differentially expressed fungal genes in *P*. *koraiensis*

As previously mentioned, the fungal transcripts expressed in infected seedlings under drought stress, which represented the dieback progression, were filtered out from the infected seedling RNA-seq data utilizing the RNA-seq *de novo* assembly of *C*. *ferruginosum* (Fig. 4). The resulting transcriptomes were studied to identify the most prominent fungal transcripts expressed in infected seedlings under drought conditions. Hierarchical cluster analysis of the *C*. *ferruginosum* unigenes was conducted and a cluster of 7,278 unigenes of *C*. *ferruginosum*, which were distinctively expressed in the infected seedling (Reads Per Kilobase per Million mapped reads, RPKM > 1), were identified. Most of these 7,278 unigenes encode secretory proteins that lack clearly characterized homologs in other sequenced organisms. These unigenes showed a distinct expression pattern under drought stress and thus support a major role for the encoded proteins during the interaction with the pine tree. Additionally, a total of 99 differentially regulated *C*. *ferruginosum* unigenes overlapped with the PR genes available in the Pathogen-Host Interaction (PHI) database (www.phi-base.org). Among these 99 unigenes, 10 of unknown function were noted to be up-regulated.

### Characterization of the differentially expressed *P*. *koraiensis* genes as a result of drought stress post *C*. *ferruginosum* infection

**Figure 5:**
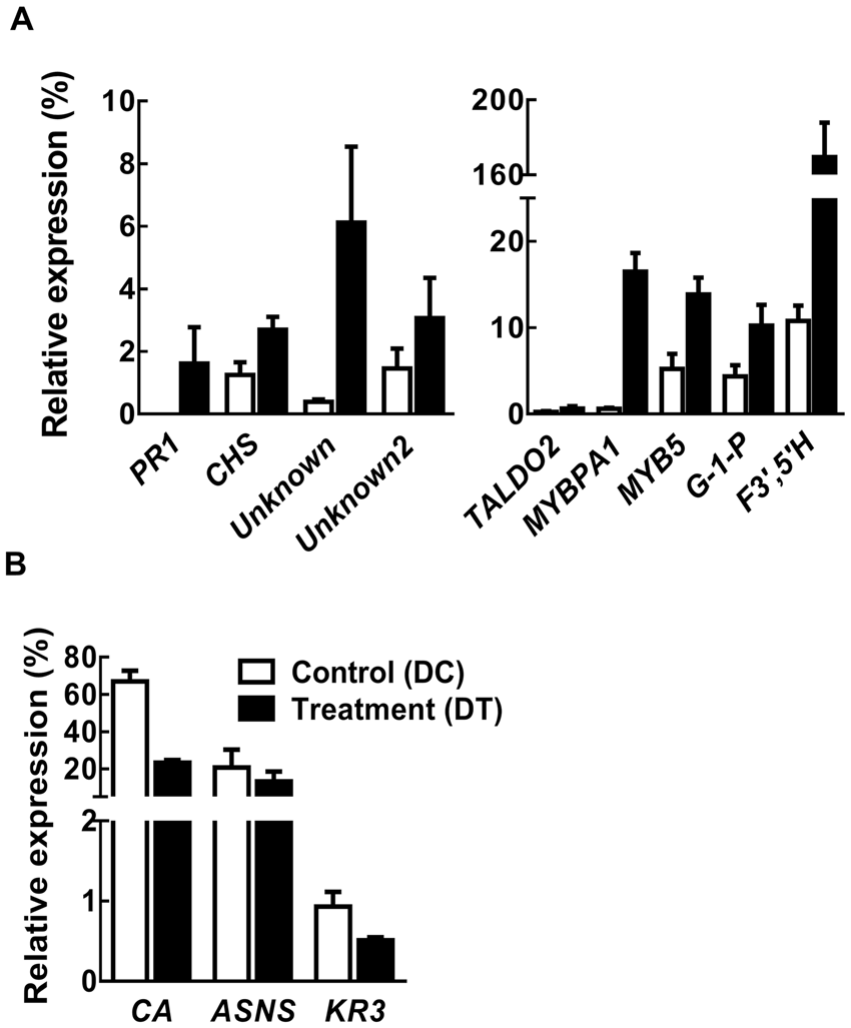
Expression analysis of selected genes of *Pinus koraiensis* inoculated with *Cenangium ferruginosum* and exposed to drought stress. (**A**) Expression pattern of stress-related and biosynthetic genes. (**B**) Expression pattern of defense-related genes. Control (DC) indicates needles of infected pine seedlings grown under optimal water supply, whereas treatment (DT) indicates needles of infected pine seedlings exposed to drought stress for 42 d. The relative expression values were obtained by qRT-PCR normalized against pine *ACTIN g*ene and are shown as the averages ± standard error, obtained from three biological replications.

Gene ontology (GO) analysis of the differentially expressed genes was performed to have an idea of the biological processes, molecular functions, and cellular components that were affected under the water deficit condition post *C*. *ferruginosum* infection. The biological processes broadly affected included stress responses and defense responses. The biosynthetic genes, for example, transaldolase (*TALDO2*), glucose-1-phosphatase (*G-1-P*), chalcone synthase (*CHS*), *MYB5*, and *MYBPA1*, were induced by drought stress in infected seedlings (Fig. 5). *TALDO2* is involved in lignin biosynthesis and *G-1-P* is biosynthetic gene involved in glycolysis. MYBPA1 is involved in the biosynthesis of proanthocyanidins (oligomeric flavonoids), which are among other chemical players in plant defense against pathogens and predators^17,18^.

Contrastingly, however, the defense-related genes, for example, carbonic anhydrase (*CA*), asparagine synthetase (*ASNS*), and R (resistance) gene homologue (*KR3*), were repressed by drought stress. Furthermore, gene involved in biosynthesis of secondary metabolites via the flavonoid pathway (*F3′*,*5′H* encoding flavonoid 3′,5′-hydroxylase) was enhanced. Along with *F3′*, *5′H*, another flavonoid biosynthetic genes *CHS* was remarkably up-regulated in drought stressed seedling post *C*. *ferruginosum* infection. Several genes associated with diverse cellular components, such as the cytoplasm, endomembrane, proteasome complex, and mitochondrion, were significantly induced. In addition, two unknown genes were highly up-regulated by drought stress. Noticeably, a pathogenesis-related marker gene, *PR1*, also exhibited considerable induced expression. Contrastingly, the expression of the defense-related genes *CA*, *ASNS*, and *KR3* encoding a TIR-NBS-LRR protein, was down-regulated. *CA* and *KR3* were significantly down-regulated in the drought-exposed infected seedlings as compared to the control.

We validated the RNA-seq results by qRT-PCR analysis. Expression of few selected genes from pine trees was validated and a good correlation existed between the RNA-seq results and qRT-PCR profiling (Fig. 5).

### Drought stress triggers *Cenangium*-dieback appearance in seedlings

Expectedly, in our results, the genes encoding putative immune receptors were induced by the infection of *C*. *ferruginosum* in pine seedlings growing under optimal water supply. However, a remarkable reduction in the levels of transcripts related to defense responses was characteristically noted in the infected seedlings that were exposed to drought stress. Of the 28,951 down-regulated unigenes in drought experiencing infected seedlings, 154 unigenes belonged to the receptor-like kinase/protein classes.

### Gas chromatography-mass spectrometry (GC-MS) analysis for identification of terpenoids in *P*. *koraiensis*

Accurate and specific method coupling GC-MS was developed for the identification of terpenoids in the hexane extracts of *P*. *koraiensis* samples. Employing the same, we could identify different terpenoids as shown in Tables 2 and 3. The major terpenoidal compounds identified from non-infected pine needles comprised lidocaine, trimecaine, N-ethyl-N-(2-cyanoethoxyethyl)−3-methyl-4-formylaniline, (E)−6-mesitoyloxy-4-methyl-5-hexenal, and fridela-7-ene. The terpenoidal compounds identified from non-infected stems were α-muurolol, thunbergol, 1H-naphtho [2,3-c] pyran-5, and daniellic acid. A clear difference in the content and level of terpenoids were noted between the tissues collected from non-infected seedlings growing under well-watered and water deficit conditions. Exact values are tabulated in Table 2. Significant levels of lidocaine (10.83%) and nonacosane-10-o1 (component of wax, 54.49%) were noted only in case of needles from drought stressed seedlings (Table 2). Two compounds - trimecaine and friedela-7-ene - were only detected in the well-watered seedlings (Table 2).

**Table 2:** Metabolic profiling of *Pinus koraiensis* needle and stem tissues.

Source	Name	CO^a^ (area %)	DR^b^ (area %)
Needle	lidocaine	ND^c^	10.83
	trimecaine	20.52	ND
	phytol	4.39	1.94
	N-ethyl-N-(2-cyanoethoxyethyl)-3-methyl-4-formylaniline	6.13	3.19
	(E)-6-mesitoyloxy-4-methyl-5-hexenal	19.36	7.77
	nonacosan-10-o1	ND	54.49
	friedela-7-ene	8.73	ND
Stem	δ-cardinene	3.57	ND
	α-muurolol	0.72	7.02
	T-muurolol	1.08	1.11
	thunbergol	6.89	0.33
	1H-naphtho[2,3-c]pyran-5	12.09	27.88
	daniellic acid	45.86	ND
	kaurenoic acid	1.72	3.51

The percentage peak area indicates the amount of individual metabolite present in the particular fraction.

^a^CO indicates pine tree data in *P*. *koraiensis* grown under well-watered condition for 42 d.

^b^DR indicates pine tree data in *P*. *koraiensis* exposed to drought for 42 d.

^c^ND indicates no detection.

**Table 3:** Metabolic profiling of *Pinus koraiensis* needle and stem tissues, inoculated with *Cenangium ferruginosum*.

Source	Name	DC^a^ (area %)	DT^b^ (area %)
Needle	3-carene	9.84	ND^c^
	terpinolene	2.16	4.70
	bornyl acetate	2.16	3.53
	β -caryophyllene	15.67	10.59
	γ- cardinene	4.05	4.23
	δ- cardinene	9.11	9.27
	paustrol	3.31	4.00
	phytol	5.21	4.32
Stem	α-terpineol	4.89	3.82
	isolongifolene	4.74	3.45
	δ- cadinene	5.58	6.50
	ethyl palmitate	7.06	7.17
	ethyl heptadecanoate	9.12	4.96
	ethyl linolenate	18.62	15.10

The percentage peak area indicates the amount of individual metabolite present in the particular fraction.

^a^DC indicates pine tree data in *P*. *koraiensis* grown under well-watered condition for 42 d post *C*. *ferruginosum* infection.

^b^DT indicates pine tree data in *P*. *koraiensis* exposed to drought for 42 d post *C*. *ferruginosum* infection.

^c^ND indicates no detection.

Contrastingly, in the stem, drought resulted in an increase of α-muurolol, 1H-naphtho [2, 3-c] pyran-5, and kaurenoic acid levels (Table 2). Reductions were detected in the levels of δ-cadinene, thunbergol, and daniellic acid, where δ-cadinene and daniellic acid were below detection in the drought samples (Table 2).

In contrast to the non-infected seedlings, the profile/levels of terpenoids in the infected seedlings were remarkably different under both the optimal and water deficit conditions. Needles harvested from infected and well-watered seedlings showed higher levels of 3-carene (9.84%) and β-caryophyllene terpenoid, where former was below detection in the infected seedlings experiencing drought (Table 3). However, the levels of terpinolene, bornyl acetate, and paustrol were relatively low in the well-watered seedlings. Stem tissues from the same seedlings showed the presence of α-terpineol, isolongifolene, δ-cadinene, ethyl palmitate, ethyl heptadecanoate, ethyl linolenate, and ethyl oleate (Table 3). Also, in the infected seedlings, the status of water availability resulted in a noticeable difference in the composition and content of terpenoids. The compound 3-carene, which was detected in the needles of infected seedlings growing under optimal water supply, was absent in needles of drought-stressed infected seedlings. Further, the level of β-caryophyllene was noticeably reduced. On the other hand, other terpenoids, such as terpinolene, bornyl acetate, and paustrol were slightly increased in infected needles experiencing drought. Similarly, in stem tissues from infected seedlings, δ-cadinene showed a slight increase under drought conditions. However, the levels of α-terpineol, isolongifolene, ethyl heptadecanoate, and ethyl linolenate decreased under drought stress (Table 3).

### GC-MS analysis for identification of fatty acids in *C*. *ferruginosum*

To reveal the effects of *P*. *koraiensis* extracts and artificial drought stress (PEG) on the fungus, we examined the fatty acid content extracted from *C*. *ferruginosum* through GC-MS analysis. The relative percentages of fatty acid esters are presented in Table 4. The levels of ethyl palmitate, ethyl heptadecanoate, ethyl linoleate, ethyl oleate, and ethyl stearate were almost similar in case of *C*. *ferruginosum* exposed to PEG-mediated drought and distilled water (used as a control). However, *C*. *ferruginosum* treated with the metabolic extracts of healthy and drought-stressed pine seedlings showed a noticeable decline in the levels of ethyl palmitate, ethyl heptadecanoate, ethyl linoleate, and ethyl oleate fatty acids as compared to the control. The compound 1,5-diethoxyanthraquinone was only detected in the samples where *C*. *ferruginosum* was treated with the metabolic extracts of drought-stressed pine seedlings (26.63%; Table 4).

**Table 4:** Metabolic profiling of *Cenangium ferruginosum*.

Source	Name	PC^a^ (area %)	PT^b^ (area %)
*C*. *ferruginosum*	ethyl palmitate	22.90	24.72
	ethyl heptadecanoate	15.95	13.64
	ethyl linoleate	37.83	39.22
	ethyl oleate	19.93	19.57
	ethyl stearate	3.39	2.85
**Source**	**Name**	**MC** ^**c**^ **(area %)**	**MT** ^**d**^ **(area %)**
*C*. *ferruginosum*	ethyl palmita	22.25	16.07
	ethyl heptadecanoate	13.86	10.65
	ethyl linoleate	40.48	29.18
	ethyl oleate	20.96	14.80
	ethyl stearate	2.44	2.66
	1,5-diethoxyanthraquinone	ND^e^	26.63

The percentage peak area indicates the amount of individual metabolite present in the particular fraction.

^a^PC indicates *C*. *ferruginosum* data in *C*. *ferruginosum* treated with distilled water for 6 h.

^b^PT indicates *C*. *ferruginosum* data in *C*. *ferruginosum* treated with 5% PEG for 6 h.

^c^MC indicates *C*. *ferruginosum* data in *C*. *ferruginosum* treated with metabolic extract of healthily growing *P*. *koraiensis* for 6 h.

^d^MT indicates *C*. *ferruginosum* data in *C*. *ferruginosum* treated with metabolic extract of drought-stressed *P*. *koraiensis* for 6 h.

^e^ND indicates no detection.

### Phytohormonal analysis

**Figure 6:**
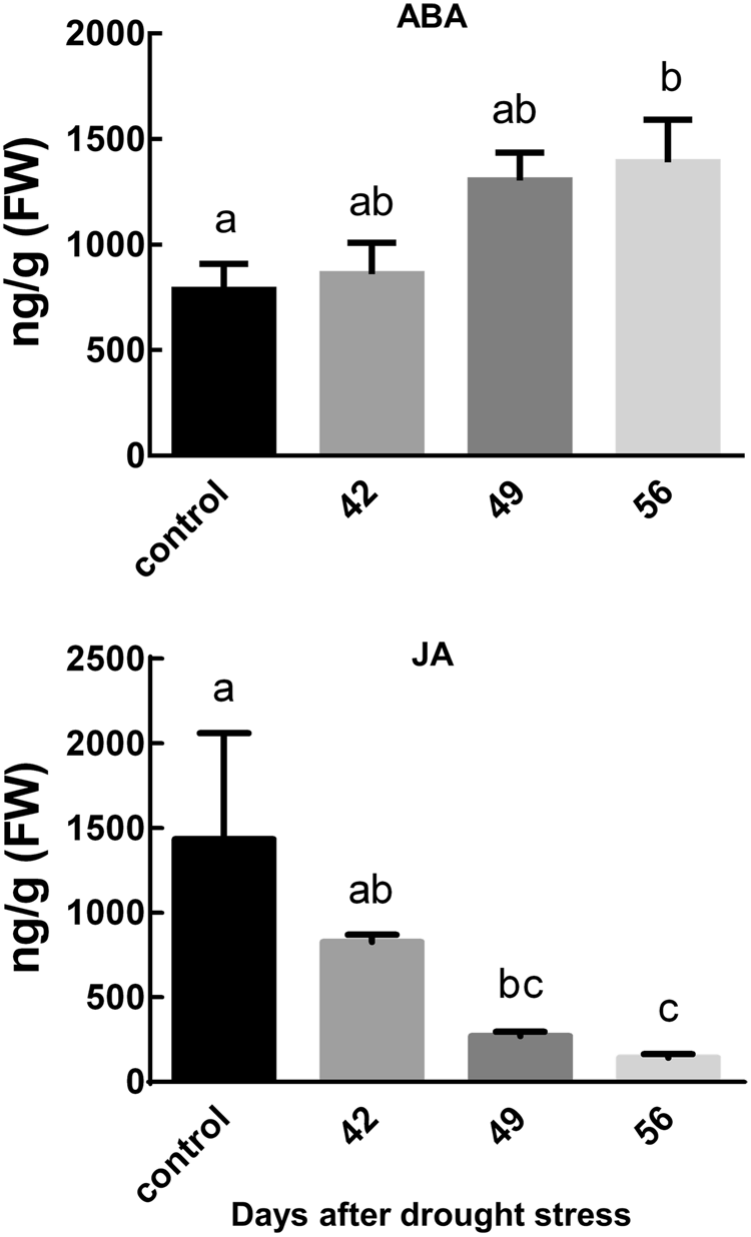
Effects of drought stress on the hormonal status of *Pinus koraiensis* infected with *Cenangium ferruginosum*. The levels of ABA and JA were measured with progressing drought (42, 49, and 56 d of drought stress) post *C*. *ferruginosum* inoculation. The control indicates the data of the pine trees grown under well-watered conditions for 42 d post *C*. *ferruginosum* inoculation. The given data are the average value ± standard error, obtained from three biological replications. The same letters indicated no significant difference.

We noted a significant alteration in endogenous phytohormonal levels (*P < *0.05) in pine seedlings infected with *C*. *ferruginosum* and simultaneously experiencing drought. The levels of ABA increased with progressing drought in infected pine seedlings, with highest levels in 56 d drought samples (Fig. 6). Contrastingly, JA levels were significantly decreased under similar conditions as compared to the control (infected seedlings with optimal water supply).

### Non-targeted metabolic profiling in *P*. *koraiensis* using liquid chromatography-mass spectrometry (LC-MS)

**Figure 7:**
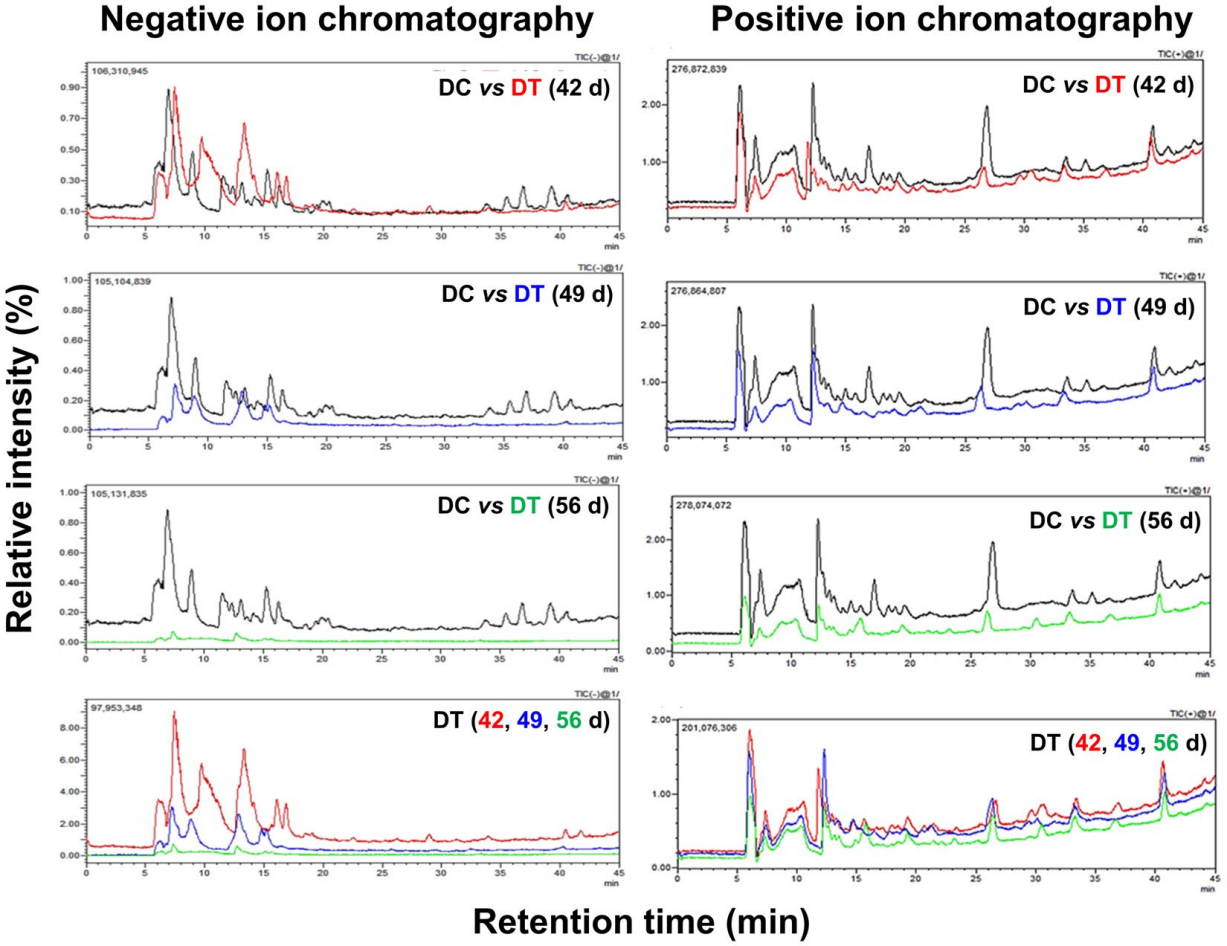
Representative LC-MS negative/positive ion chromatograms of *Pinus koraiensis* stem extracts. DC represents the extracts of infected pine seedlings grown under optimal water supply for 42 d and DT represents the extracts from infected pine seedlings treated with drought stress for 42 (red), 49 (blue), and 56 (green) d.

The effect of drought stress on the secondary metabolite profile of infected pine seedlings was deciphered through LC-MS analysis. Elemental composition analysis was carried out by accurate data combined with the natural abundance of the isotopic ion. In addition, stable isotope labeling was used for this purpose. Both negative and positive ion mode of electrospray ionization (ESI) was selected for MS analysis. Extensive structure information about most phenolic acids and flavonoids present in the samples were obtained by the negative and positive ion modes, respectively. The negative ion chromatograms for phenolics present in the extracts of pine trees are shown in Fig. 7. Herein, the chromatographic peaks were compared from both the well-watered and drought-stressed infected seedlings by comparing the retention times (*T*_*R*_), UV, and MS data (Fig. 7). A clear decline in the mass area of most peaks was observed in the infected seedlings exposed to drought as compared to the control (infected seedlings with optimal water supply). Similarly, we noted a decline in flavonoids, as shown in the positive ion chromatograms in the infected seedlings experiencing drought as compared to the infected seedlings watered optimaly).

## Discussion

The detrimental consequences and increasing frequency of *Cenangium*-dieback makes understanding the biology of this disease important in the development of preventive strategies. Recently, the high-throughput omics data attributing the plants’ interaction with *Botrytis cinerea* (another important necrotrophic plant fungal pathogen) has been suggested to be amply useful for genetic improvement of plants against gray mold disease^19,20^. This further highlights the usefulness and pressing need of a detailed study revealing the molecular complexities behind the progression of die-back disease in pine. Necessiated by the above arguments, our study focused on developing a profound knowledge of the molecular interaction between *C*. *ferruginosum* and *P*. *koraiensis*, with an aim to reveal the progression of *Cenangium*-dieback in plausible molecular terms.

*C*. *ferruginosum*, which otherwise is a pine endophyte, turns pathogenic with the advent of stressful conditions, such as drought^3^. Under drought stress, the asymptomatic biotrophic fungus exhibits a peculiar hemibiotrophic life cycle, which is marked by dieback symptoms – destructive plant necrosis. Under conditions favorable for pine trees, the asymptomatic biotrophic phase of *C*. *ferruginosum* lasts for a long time without any dieback symptoms. We thus simulated the disease conditions by infecting healthily growing pine seedlings with *C*. *ferruginosum* and withholding water post infection to cause drought stress. Concurrence of pathogen infection and drought for 42 d or more showed necrotic dieback symptoms unlike the watered seedlings, suggesting the successful simulation of the disease progression. After establishing the disease symptoms, we next studied the molecular interaction between the host and pathogen in terms of transcriptomic, metabolomic, and hormonal alterations.

The development of a disease largely depends on the molecular interplay between the pathogen and its host, which involves substantial transcriptional changes in both organisms^21^. Consequent to a complex interaction between host and pathogen, the gene expression changes in both organisms are highly regulated and dynamic^21^. To study such dynamic alterations in transcriptomes associated with both host and pathogen, the concept of ‘dual RNA-seq’ was introduced^22^ and has already been used comprehensively in several previous studies^21,23^. Using a similar approach in this study, we could identify transcripts that were significant during the biotrophic interaction between *P*. *koraiensis* and *C*. *ferruginosum*: 86 and 99 million reads mapped to plant and fungal gene models, respectively. From the gene expression results, it becomes obvious that under well-watered conditions the pine tree’s defense against *C*. *ferruginosum* remained activated, although insufficiently to suppress the growth of *C*. *ferruginosum* completely, and the defense-related pine genes *CA*, *ASNS*, and *KR3*, were highly expressed in the control (infected seedlings with optimal water supply). Similarly, with the advent of drought, where the unaffected fungal gene expression enables the fungus to overgrow, pine tree tries its best to counteract it. The overexpression of *MYBPA1*, *MYB5*, belonging to R2R3-MYB transcription factor family, member of which have already been shown to play important role in plant’s tolerance against the necrotrophic plant pathogen *Botrytis cinerea*^24,25^, is an indicative of the triggered defensive mode. However, plant still fails to mount an effective defense against the overgrowing pathogen as the other important defense-related pine genes, including *CA*, *ASNS*, and *KR3*, were down-regulated. CA plays a role in plant immunity through oxidative stress protection^26^. ASNS plays a key role in nitrogen remobilization, which is important and modulated in parallel to defense responses^27^. KR3 plays an important/primary role in detection of pathogens and elicitation of defense responses. This might have resulted in an oxidative burst and reduced elicitation of defense responses. Additionally, the various transmembrane and intracellular receptor-like kinases/proteins (putative immune receptors) that remained induced under optimal water supply declined remarkably under drought stress. Altogether, this possibly resulted in decreased immunity of the pine trees, which enabled the unaffected fungus to overgrow and cause dieback disease.

Corroboratively, the RNA-seq data showed a clear increase in the percentage of reads mapping to *C*. *ferruginosum* in infected pine seedlings exposed to drought (7.49% and 7.59% in control and treatment, respectively), which suggested that more *C*. *ferruginosum* genes were expressed when plants experienced drought post infection. Overall, it is amply clear that the interaction between *C*. *ferruginosum* biotrophic hyphae and pine tree cells continues for a long time under optimal conditions and the plant succeeds in keeping a check on the fungal growth. In other words, *C*. *ferruginosum* and pine trees establish a mutualistic association, which is also marked by the absence of the expression of the marker gene indicative of pathogen attack – the pine *PR1* gene. Similar observations are reported previously in several biotrophic pathogens^28,29^. However, an episode of drought weakens plant immunity, thereby enabling the endophytic fungus to overgrow, become a pathogen, and cause dieback. This is also marked by the increased expression of the pine *PR1* gene in infected seedlings experiencing drought. Finally, our first of its kind transcriptomic analysis thus presents an inventory of possible candidate genes in pine that can be targeted for genetic improvement of pines specifically against *Cenangium*-dieback disease. Certainly, it first requires extension of this pioneering work through more directed research in the future.

The unavailability of complete genome sequences of both *C*. *ferruginosum* and *P*. *koraiensis* limited the possibility of more detailed analyses/inferences from the transcriptomics results. We thus extended our study to also reveal related consequences that may account for the susceptibility of pine trees for *Cenangium*-dieback under drought conditions. Precisely, we chose to screen the alterations in the levels of terpenoids, flavonoids, and phenolics, which are well known antimicrobial compounds synthesized in pine species. Indeed, a significant difference in the terpenoid production/levels was noted in the pine seedlings growing under well-watered and drought conditions post *C*. *ferruginosum* infection. The concentrations of most of the terpenoids, such as 3-carene, β-caryophyllene, phytol, α-terpineol, isolongifolene, ethyl heptadecanoate, and ethyl linolenate, decreased significantly in the needle/stem tissues of *C*. *ferruginosum-*infected pine seedlings exposed to drought stress. In previous studies, drought stress was noted to increase the concentrations of terpenoids in wood and needles of conifers^30^. Interestingly, however, our study suggests that the combination of pathogen-infection and drought had a contrasting effect on the terpenoid profiles. We thus hypothesized that the lowered levels of terpenoids weakened plant defenses and made it more prone to disease. Along with terpenoids, the levels of flavonoids and phenolic acids, which constitute the major bioactive compounds in plants and are important in defense against pathogens^15,16^, were also significantly reduced in drought-exposed *C*. *ferruginosum-*infected seedlings. Overall, a significant decrease in the levels of secondary compounds in the constitutive resin was noted in drought stressed pine seedlings (both non-infected and infected). We thus believe that drought stress triggered a reduction of these compounds and decreased the immunity of pine trees against *C*. *ferruginosum*, which lead to the development of *Cenangium*-dieback in pine seedlings.

Phytohormones play a critical role in plant development against many biotic and abiotic environmental factors. Different phytohormones and associated signaling may interact in a synergistic or an antagonistic manner, which results in a customized developmental response. Many previous studies have strikingly noted that disease resistance and drought tolerance are often antagonistic^31^. These effects were attributed to the activation of the ABA pathway under drought stress^32,33^, which through antagonism suppresses SA-, JA-, and ethylene-mediated signaling pathways and thus the disease resistance in plants^31^. In agreement with these reports, we too noted an increment in ABA levels and a corresponding decline in JA levels with progressing drought. JA is believed to play a significant role in plant defenses against necrotrophic and biotrophic fungal pathogens^4,20^. We therefore propose that reduced levels of JA weakened the ability of pine seedlings to mount an effective defense against *C*. *ferruginosum*, which resulted in the development of *Cenangium*-dieback disease.

Drought stress is well known to decrease the host resistance and/or increase nutritional value of the plants, which accelerates fresh pathogen infections and disease outbreaks^30,34^. However, in case of *Cenangium*-dieback, it is important to note that the *C*. *ferruginosum* that colonizes the host plant is already present as an endophyte and causes disease only after a drought episode. We thus questioned whether drought stress that reduces the immunity of pine seedlings also affects the pathogenicity of *C*. *ferruginosum*. If yes, how does the pathogen develop the disease? Although the gene expression analysis suggested that there was no adverse effect of drought on *C*. *ferruginosum* growth and pathogenicity, we confirmed it at the level of fatty acid contents in the fungus. *C*. *ferruginosum* grown in metabolic extracts of well-watered, as well as drought-stressed pine seedlings, and PEG were studied to look for any change in fatty acids. Interestingly, no remarkable difference/effect on the contents of the studied fatty acids was noted. This suggested that environmental drought reduces only the pine’s immunity, and does not impose any indirect negative effect on the pathogenicity of the fungus. This unaffected virulent state of the pathogen combined with the drastically reduced ability of the plant to mount a defense facilitated the development of *Cenangium*-dieback in drought-stressed pine seedlings.

**Figure 8:**
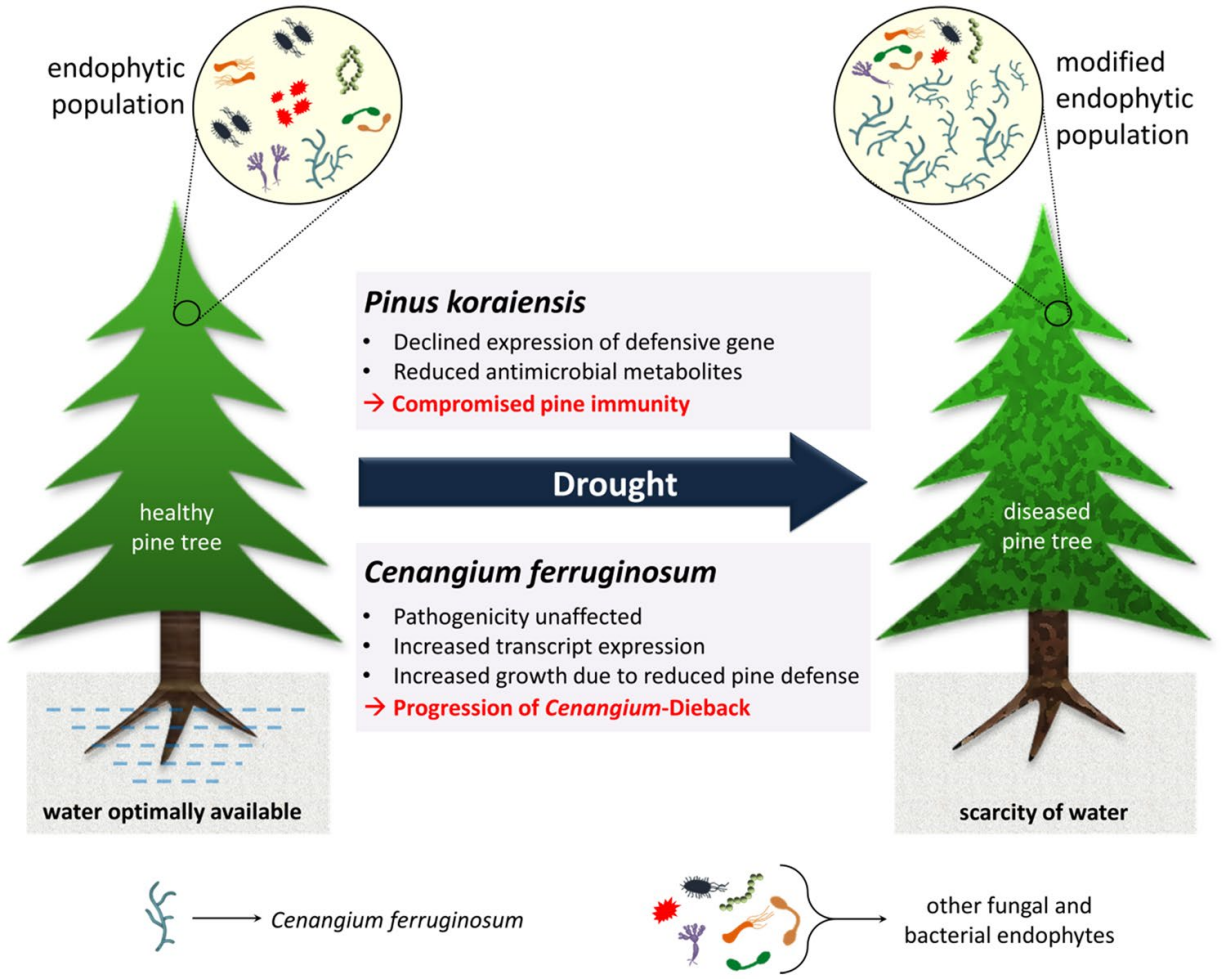
Summary of the mechanism of drought-induced susceptibility for *Cenangium ferruginosum* in *Pinus koraiensis*.

In conclusion, we present a comprehensive study entailing the metabolomic (e.g., resin, essential oil, flavonoid, and phenolic acid) and transcriptomic changes possibly responsible for conversion of an endophyte to a pathogen under drought stress. In so doing, we bring forth the molecular mechanisms that (1) *P*. *koraiensis* uses to control the development and virulence of *C*. *ferruginosum* under optimal conditions, and (2) lead to the development of disease under drought stress (Fig. 8). Hitherto, the information about transcriptional alterations entailing host-pathogen interaction regarding *Cenangium*-dieback was insufficient. Our study, as it reports a transcriptomic analysis of the interaction of *C*. *ferruginosum* and pine seedlings under drought stress, will vastly expand our knowledge of *Cenangium*-dieback disease. It will open the window for more detailed analysis of disease progression in the future.

## Methods

### Biological material, disease/stress assays and sampling

*C*. *ferruginosum* was isolated from infected branches of *P*. *koraiensis* growing in wild^2^. The fungus was then cultured on MSA medium containing 3% malt extract, 0.3% soya peptone, and 1.5% agar (Difco Co., Detroit, MI, USA) at 20 °C for 4 weeks in the dark before usage in this study.

To study the consequences of pathogen infection on the host, pine seedlings were infected with *C*. *ferruginosum* and analyzed under both well-watered and water deficit conditions. Twenty 5-year-old pine seedlings were grown in pots under greenhouse conditions. Each of the 20 healthy pine seedlings was spray-inoculated with 10 mL of a *C*. *ferruginosum* spore suspension (10^4^ ascospores mL^−1^ in H_2_O, 87% ascospore germination rate) and then maintained at a humidity of ≥90% for 3 d to induce spore germination and infection. Fifteen seedlings were then subjected to drought stress for 42, 49, and 56 d allowing the use of 5 biological replicates for each condition. The remaining 5 seedlings were irrigated twice a week with normal water supply to serve as a control. A similar setup of 10 seedlings was maintained without inoculation of *C*. *ferruginosum* and grown with and without drought stress for 42 d. Twigs and needles were harvested from the second branch from the base of the tree crown and then immediately snap-frozen, ground in liquid nitrogen, and stored at −80 °C until further use.

To investigate the effects of the metabolic extracts of pine seedlings on the pathogen, *C*. *ferruginosum* was treated with pine extract isolated from drought-stressed, without *C*. *ferruginosum* inoculation samples. As a control, pine extract isolated from healthy (well-watered) seedlings without *C*. *ferruginosum* inoculation was used. *C*. *ferruginosum* was first grown in MSB (broth containing 3% malt extract and 0.3% soya peptone) for 7 d at 23 °C and 150 rpm. Subsequently, 2.5 mL of MSB culture was replaced with 2.5 mL of 5,000 ppm pine extract. Similarly, to check the effects of drought stress, instead of pine extract, 2.5 mL of 5% PEG 6000 (Sigma-Aldrich, St. Louis, MO, USA) was used. Culture supplemented with 2.5 mL of H_2_O served as a control. After the aforementioned treatments, all the tubes were maintained at 23 °C and 150 rpm and the samples were harvested after 1 (for RNA-seq) and 6 h (for metabolite analysis). Samples were then frozen in liquid nitrogen and stored at −80 °C until further analysis. For growth rate estimation, hyphae of *C*. *ferruginosum* were grown in MSA media supplemented with various concentrations of PEG (5, 10, 15, and 20%).

### RNA extraction and sequencing

Total RNA from infected pine needles was isolated using the CTAB method^35^. Total RNA from mycelia of *C*. *ferruginosum* grown in potato dextrose broth (PDB) was extracted using the CTAB method^36^ and a Qiagen RNeasy Plant Mini Kit (Qiagen, Valencia, CA, USA). The quality of RNA, which was determined by the RNA integrity number (RIN), was assessed by an Agilent 2100 Bioanalyzer using the RNA 6000 Nano Chip (Agilent Technologies, Santa Clara, CA, USA). The quantity of total RNA was determined by Infinite 200 PRO NanoQuant Spectrophotometer (Tecan, Männedorf, Switzerland).

Transcriptome libraries were prepared following Illumina’s TruSeq RNA protocol (San Diego, CA, USA), using 1–2 μg of total RNA. Poly(A)^+^ RNA was isolated using AMPure XP beads (Beckman Coulter, Brea, CA, USA) and fragmented with the Ambion Fragmentation Reagents Kit (Ambion, Austin, TX, USA). cDNA synthesis, end-repair, A-base addition, and ligation of the Illumina indexed adapters were performed according to Illumina’s protocol. Libraries were size-selected for 250–300 bp cDNA fragments on a 3% Nusieve 3:1 agarose gel (Lonza, Basel, Switzerland), recovered using QIAEX II gel extraction reagents (Qiagen), and PCR-amplified using Phusion DNA polymerase (New England Biolabs, Ipswich, MA, USA) for 14 PCR cycles. The amplified libraries were purified using AMPure XP beads. Library quality was measured on an Agilent 2100 Bioanalyzer for product size and concentration. Paired-end libraries were sequenced with the Illumina HiSeq. 2000, (2 × 100 nucleotide read length). Reads that passed the chastity filter of Illumina Base Call software were used for subsequent analysis.

### Transcriptome analysis

Transcriptome analysis was performed using the Tuxedo protocol. Sequences were mapped against the Human reference genome (Ensembl release 69) using TopHat v2.0.9 with default options for paired-end sequences and transcript expression was estimated using the Cufflinks program v2.1.1 with genome annotation data (Ensembl release 69).

### Fatty acids analysis using GC-MS

Lyophilized needles and twigs (0.3 g) from each pine seedling were placed in a glass vial where 1 mL of 2.5% H_2_SO_4_ (v/v) in methanol and 32 µg of internal standard (heptadecanoic acid) were added. Samples were heated to 80 °C for 1 h using a water bath and then cooled down to room temperature. The mixture of 1.5 mL pentane and 4.5 mL 0.9% NaCl (w/v) was added to extract fatty acid methyl esters (FAME). Samples were then shaken vigorously and centrifuged at 14,000 rpm for 5 min at 4 °C to facilitate phase separation. The upper phase (pentane-containing FAME) was then transferred to an injection vial. Fatty acid analysis was performed by GC with a detector. The samples were analyzed using an Agilent 7890 A GC equipped with a Econo-Cap^TM^EC^TM^-Wax capillary column (15 m × 0.53 mm × 1.2 µm; Alltech Associates, Deerfield, IL, USA) and flame ionization detector. For GC-MS detection, an electron ionization system with ionization energy of 70 eV; ion source temperature of 250 °C was used. Helium was the carrier gas, at a flow rate of 1 mL min^−1^; 1:50 split ratio. Injector and detector MS transfer line temperature was programmed to 40 °C, held 2 min, raised to 250 °C at 10 °C min^−1^, and held for 10 min.

### Phytohormone analysis

Two apical twigs per pine seedling were processed for the phytohormones analysis. Phytohormones were extracted and purified according to the protocol described previously^37^. Lyophilized samples (0.25 g) were extracted with 1.25 mL of extraction solution comprising methanol/water (80/20, v/v). Extracts were kept at −20 °C for 1 h, and then centrifuged at 20,000 rpm for 15 min at 4 °C. The pellets were re-extracted in an additional 1.25 mL of the same solution for 30 min. Collected supernatants were filtered through Sep-Pak C18 Plus (Waters, Milford, MA, USA) to remove interfering lipids and pigments, and evaporated at 40 °C using a speed vacuum. Residues were then dissolved in 1 mL of methanol/water (20/80, v/v) solution using an ultrasonic bath (Emerson, St. Louis, MO, USA). Samples were filtered through 13 mm diameter nylon syringe filters (0.45 μm) (Sigma-Aldrich) and the volume was adjusted to 1.5 mL using the extraction solution. Calibration curves were constructed using different concentrations of internal standards (Sigma-Aldrich) – ABA (50, 100, 200, 400, and 600 ng mL^−1^) and JA (10, 20, 40, 80, and 120 ng mL^−1^) – and recoveries ranged over 99%. Four biological replicates for each condition/sample were quantified. LC-MS conditions followed for the analyses of phytohormones are described below.

### Metabolite analysis using LC-ESI-MS/MS

Secondary metabolites were extracted from pine seedlings according to the protocol described by Moco, *et al*.^38^. Four biological replicates were used for each condition/sample. Tissue samples (0.25 g) were extracted in 1.5 mL pure methanol (final methanol concentration in the extract was ~75%). Subsequently, 1 mL of 0.1% tert-butylhydroquinone and 0.4 mL of 6 M HCl (in methanol solution) were added and the extracts were heated on a heat block at 90–95 °C for 1 h. Finally, after adding 2 mL of pure methanol, the samples were sonicated (50–60 Hz) for 15 min and then centrifuged at 13,000 rpm for 10 min at room temperature. Supernatants were collected and filtered through 0.45 μm inorganic membrane filter (Sigma-Aldrich), fitted into a disposable syringe, and transferred to a glass vial.

LC-MS-8040 tandem quadrupole mass spectrometer (Shimadzu, Kyoto, Japan) equipped with an LC-30AD binary pump was used for the untargeted metabolic profiling. Composition of the mobile phase was degassed formic acid:water (1:103, v/v; eluent ‘A’) and 100% acetonitrile (eluent “B”). The chromatographic separation was accomplished using an ACE UltraCore 2.5 SuperC18 column (150 mm × 4.6 mm, 2.5 µm; Aberdeen, Scotland, UK) at 40 °C and eluted at a flow rate of 0.5 mL min^−1^. The elution gradient consisted of 0.01–2 min, 0–40%; 2–5 min, 40–60%; 5–13 min, 60–100%; 13–15 min. Subsequently, the column was washed with 100% B for 15 min before the next injection. The injection volume was 5 µL. Ionization was performed using an electrospray ionization source, and masses were detected in both positive and negative mode.

### Statistical analyses

Values were represented as the mean of 3–6 biological replications. An analysis of variance (ANOVA) followed by Duncan’s multiple range test (DMRT) was conducted to determine the significant differences among means at a level of *P* = 0.05 using the statistical software SAS (Portable version 9.1.3, Cary, NC, USA). Graphs were prepared using GraphPad Prism 6 (San Diego, CA, USA).

## Electronic supplementary material

Supplementary Figure S1
