# Supplementary material for: Drought-induced susceptibility for Cenangium ferruginosum leads to progression of Cenangium-dieback disease in Pinus koraiensis
Source: Sci Rep. 2018 Nov 6;8:16368. doi: 10.1038/s41598-018-34318-6 (PMC6219526; doi:10.1038/s41598-018-34318-6)
Supplement: Supplementary file 1 — Supplementary Figure S1 [file 41598_2018_34318_MOESM1_ESM.pdf]

Drought-induced susceptibility for *Cenangium ferruginosum* leads to progression of *Cenangium*-dieback disease in *Pinus koraiensis*

Minji Ryu[1,ǂ], Ratnesh Chandra Mishra[1,ǂ], Junhyun Jeon[1], Sun Keun Lee[3] & Hanhong Bae[1,*]

ǂ These authors contributed equally

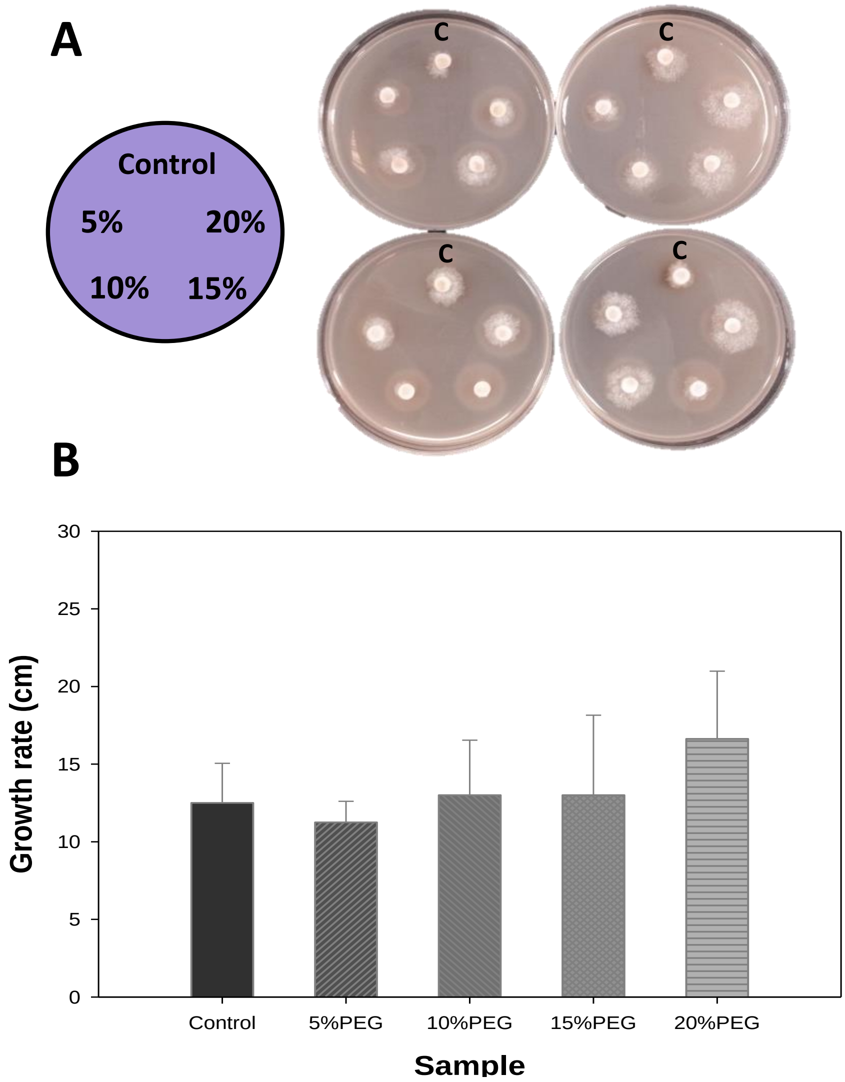

Supplementary Figure S1. Growth rate of *Cenangium ferruginosum* under artificial drought stress (polyethylene glycol). *C. ferruginosum* was grown in MSA media supplemented with various concentrations of polyethylene glycol (PEG). Morphology and growth rate were measured 7 d after culture. (A) Morphology of *C. ferruginosum* as assayed on solid media under different PEG concentrations. (B) Growth rate of *C. ferruginosum* under drought stress as measured in liquid culture.
